# Hospital and Institutional News

**Published:** 1912-05-25

**Authors:** 


### HOSPITAL AND INSTITUTIONAL NEWS.

### THE OPENING OF THE NEW PREMISES OF THE ROYAL SOCIETY OF MEDICINE.

The King and Queen opened the new buildings of the Royal Society of Medicine on Tuesday afternoon. A full description, with illustrations of the society's new home and of the Fellows' library, appears on another page. The King and Queen, who arrived at 4 o'clock, spent over an hour in the building, and in declaring it open the King said: "Medical science has revealed by experiment and trained observation new securities for life and health during recent years; and none can doubt that the improved public health is mainly due to the discoveries made by the medical profession in this and other countries; to the guidance given by that profession to civil authorities; and to the sanitary precautions against the spread of disease which they have enforced." The King further accepted from Mr. J. Y. W. MacAlister, the secretary, a model of his invention by which flexible cords for the lighting of tables are replaced by a method that secures contact through a leg of the table, which is fitted into a socket in the floor. The President, Sir Henry Morris, Dr. Arthur Latham, and Mr. H. S. Pendlebury accompanied the King and Queen on their tour of inspection. Among those present were the Lord Mayor, Sir William Church, Sir William Osler, Mr. F. W. Mott, M.D., Mrs. Scharlieb, M.D., who represented the lady Fellows, Mr. John Nachbar, M.D., editor of the Society's Proceedings, and Mr. John Belcher, the architect. It is especially noteworthy that the King took the greatest interest in the basement book-store, of which a full description, including that of an ingenious device for adapting shelves to every size of volume, is given at the end of our article.

### THE MISAPPREHENSION OF MR. J. A. PEASE, M.P.

Sir Victor Horsley last week introduced a deputation from the British Medical Association to Mr. J. A. Pease, the President of the Board of Education. Its object was to recommend the medical treatment of elementary school children through clinics instead of hospitals. Sir Victor Horsley pointed out that the hospital scheme of the London County Council was persisted in by Mr. Pease's Department, though recognised by it as involving waste and administrative chaos, and, in the opinion of the deputation, medical inefficency. Mr. Pease, in reply, said that these arguments should have been addressed not to him but to the local education authority in London, which alone could move in the matter. The Board of Education's ideal, he added, was largely that of the deputation, and the whole question of the treatment of school children was in its infancy. Quite so: from the point of view of the central or local education authorities, but emphatically not so from the point of view of the hospitals, to which the infancy of the problem is that of a prodigy whose size and menace are the dangerous element in his tender years. If the deputation's words were, from Mr. Pease's point of view, too strong, that is only because he does not appreciate the strength of the facts which they were used to illustrate.

### THE NEW SURGEON-GENERAL AND HIS WIFE.

The very gratifying announcement was made last week that his Majesty has approved of the special promotion of Col. Sir D. Bruce to the rank of Surgeon-General for his distinguished researches. The extent of those researches, and the remarkable scientific value which they always possess, are familiar to those who follow the wonderful progress of tropical medicine; and the honour with which they have been rewarded is thoroughly well deserved. Incidentally it is matter for jubilation that once a soldier has been promoted for success in saving life instead of in destroying it a precedent is set which will probably be followed frequently in years to come. It is well known that Sir David has no keener, more loyal, and more able assistant in his scientific work than Lady Bruce; and doubtless there are few distinctions which he would value so much as some public recognition of the part which she has played in his manifold successes. The suggestion may at any rate be commended to the consideration of those in authority.

### A LADY HOUSE SURGEON IN COURT.

A verdict of accidental death, with the satisfactory rider that the child received every attention at the hospital, was the result of an inquest on a boy aged five, who died soon after admission to the Royal Free Hospital as the result of burns. The mother alleged delay in admitting her son, though she was told that his burns were not severe enough to warrant it. Dr. Marian Rawlings, house surgeon to the hospital, gave evidence in support of the latter statement, and said his burns were of the first degree, and that he was kept at the hospital for several hours till risk of collapse or shock was apparently past. When she saw the boy again he was suffering from acute bronchial pneumonia, following the burns, from which he died. The father said that he thought from the way in which the boy was bandaged he should have been retained in the wards. The question at once suggests itself: Is evidence of great care in bandaging slight burns to be regarded as evidence of neglect? The care, not the burns, frightened the father, whose misapprehension forms an odd reward for the kindly treatment of his little boy at the Royal Free Hospital! This part of the case deserves to become the proverbial instance of *suggestio falsi* in a coroner's court. As for the broncho-pneumonia, this complication is of course a frequent sequela of severe burns; but in this particular case it is difficult to see how the house surgeon can fairly be saddled with blame for not foreseeing it, as the injuries seemed to be so superficial.

### THE KING'S INTEREST IN ANÆSTHETICS.

Wherever the King and Queen may be it is noticeable that they never miss an opportunity of visiting hospitals. The latest example of this occurred last Saturday, when their Majesties, towards the conclusion of their visit to Aldershot, spent the afternoon in visiting the Louise Margaret and Cambridge Military Hospitals. Shortly after 3 p.m. the Queen drove to the Louise Margaret Hospital, where her Majesty was received by Surgeon-General G. W. Robinson, Deputy Director of Medical Services, Major S. F. St. D. Green, Royal Army Medical Corps, officer-in-charge, the medical and surgical staff, and Miss E. M. Beesby, lady superintendent. Having spent some time there the Queen went on to the Cambridge Hospital, where the King joined her at 4 o'clock. A characteristically complete inspection of the institution and its organisation was made; every ward was visited. Many patients were personally addressed, and the three cases in the officers' ward received special consideration. One patient, a civilian, we may note, the Queen showed great interest in—a boy who had been lately run over by an omnibus in the neighbourhood of the hospital. The King was exceptionally interested in stovain, the favourite anæsthetic in this hospital, where he spent an hour and twenty minutes, and the Queen even longer. The King and Queen were received at the Cambridge Hospital by the Deputy Director of Medical Services, Lt.-Col. S. Hickson, Royal Army Medical Corps, officer-in-charge, the medical and surgical staff, and Miss H. W. Reid, the matron.

### THE DIVORCE COURT AS A BAR TO PUBLIC APPOINTMENTS.

The case of Dr. Goodwin, whose appointment as Medical Officer of Health to No. 8, Hounslow district, subject to the taking up of references, was ultimately cancelled by the Brentford Guardians, on the ground that his wife was suing him for a judicial separation, continues to excite lively discussions at the meetings of the Guardians. We have to add to what we wrote last week three facts. The first is that the friends of Dr. Goodwin, led by Mr. W. A. Davies, have been very active on his behalf, submitting, as we pointed out last week, that the judicial proceedings had been withdrawn some time before the day of election, and further that this withdrawal was wrongly, if accidentally, concealed from the Board. Secondly, at a meeting last Saturday Mr. Allan Hair, M.R.C.S., was appointed to the post; and lastly, and most important, that the Local Government Board has been approached by Mr. Davies and seven others, with the result that the Guardians have received a letter from the Board asking for their comments on Mr. Davies' communication. This was a lengthy document setting out the circumstances, and contending that the treatment of Dr. Goodwin by the Guardians was unfair. The matter being officially before the Board, we refrain from comment, but it will be most interesting to see what opinion, if any, the Board may be induced to give on the general question: Should divorce court proceedings be a bar to public appointments? Apart from any answer which the Board may give, and in order to test institutional feeling in this matter, we shall be glad to give publicity to our readers' views.

### A MEDICAL EDITOR ON SECRET REMEDIES.

Giving evidence before the Select Committee on Proprietary Medicines, Dr. Nestor Tirard, medical editor of the *British Pharmacopœia*, explained the attitude of the General Medical Council towards the question. He said that the compilers of the Pharmacopœia ignored proprietary medicines as far as possible, but that it was sometimes deemed advisable to introduce into the official volume a formula analogous to that of a popular medicine containing poison, with the object of safeguarding the public against changes in the proportion of the poisonous ingredient. He instanced "chlorodyne" as an example. Proceeding, Dr. Tirard said that he would like to insist, not on the full composition of a proprietary medicine being stated on the label, but on the amount of the poison being stated, as the General Medical Council was afraid that the amount sometimes varied. As our recent leading article on the "Normyl Treatment" will have led our readers to expect, Dr. Tirard, in reply to a question, said that in his experience the genesis of nearly every "patent medicine" was the prescription of a medical man. He hinted that the *British Pharmacopœia* itself was sometimes in request for the provision of a formula. It is clear from the evidence of Government officials that it is the business of no Department to supervise the sale and advertising of proprietary medicines. The Committee will meet again on June 6.

### THE NEW MENTAL HOSPITAL.

Building on the Horton Estate for the County Council's eleventh mental hospital is to be begun at once. The total cost of the new mental hospital is now estimated at £517,970, including a sum of £50,000 for equipment at the rate of £25 a bed, there being accommodation for 2,000 patients. The Mental Hospitals Committee provide in the plans, on each side of the mental hospital, for a pavilion for thirty male and thirty female phthisical or dysenteric cases; this will bring the total accommodation up to 2,060 beds. Comparing the capital expenditure, as estimated at £467,970, with the £473,738 spent on the Horton Mental Hospital, which was opened in 1902, and with the slightly larger sum incurred on the Long Grove Mental Hospital in 1907, we find that an average has been struck. The new mental hospital will take from four to five years to build and to equip. The question of building the new hospital in sections has also been discussed. This could be done by omitting from the first contract the detached buildings. But it is felt that the only reason for building the hospital in sections would be uncertainty whether full accommodation would be required from the first. The Mental Hospitals

Committee is of opinion that the wisest course is to build for the full number of patients. The estimated cost per bed works out at £235, all expenses included. It was £243 at Horton and £247 at Long Grove.

### THE LOSS OF FEVER PATIENTS TO PERTH INFIRMARY.

In accordance with an agreement between the Directors of Perth Royal Infirmary and the Perth District Committee of the County Council entered into in November 1906, the county authorities agreed that the infirmary should receive their cases of typhoid, diphtheria, erysipelas, and puerperal fever. In return for this the county authorities have paid £100 per annum to maintain the additional nursing staff necessary to treat these cases, and in addition the sum of £7 10s. for each patient admitted. This agreement has been in force ever since, and the Royal Infirmary has derived £683 from this source. The county authorities desire to terminate the agreement on Whitsunday, by which time it is expected that the County Hospital at Burghmuir will be ready for the reception of their own patients, and the Infirmary, therefore, cannot look for a revenue from this source again. This will be a loss both in experience and in finance to all concerned in the interests of the Perth Infirmary.

### PRACTICAL RESEARCH IN FEVER HOSPITALS.

There is no doubt that some patients convalescing from diphtheria continue to harbour the *Bacillus diphtheriæ* in their throats or noses for much longer periods than others, and it is equally well established that in a small percentage this period may be weeks or months after an apparent return to complete health. Hence much attention has been paid to "carrier" cases, and the routine bacterial examination of the fauces is now as essential before isolation is ended as is an anti-toxin injection when it is begun. Nor are fever hospital authorities satisfied nowadays with a single favourable report; they prefer a series of them at short intervals before they feel able to guarantee that infectivity is at an end. But this entails time, trouble, and expense, so that any simplification of the process is acceptable. On p. 197 will be found a description by Dr. Thomson Rankin of a culture medium which enables microscopical examination to be dispensed with, and an opinion as to the presence or absence of *B. diphtheriæ* on a swab to be given from naked-eye inspection of the culture tube. This is the kind of practical research which medical officers in fever hospitals have great facilities for carrying out, and such work is of high value to the community.

### AN IMMINENT DEADLOCK AT BATH.

A dispute, according to the local papers, is in progress between the board of management and the honorary staff of the Bath Mineral Water Hospital. The cause is stated to be the board's proposal to add to the medical staff three honorary assistant physicians. Matters apparently became serious when a certain committeeman circularised the governors on learning that the present honoraries took exception to the proposed addition to their number. In response to a request officially to withdraw this letter, the committee have placed their letter-writing member on the sub-committee which has been set up to deal with the question. If this be not capable of explanation, the medical staff's threatened resignation, unless the letter is withdrawn, will surprise no one. The responsibility for an explanation clearly devolves upon the committee, and it is one, we trust, that they will not attempt to shirk.

### TRANSMISSION OF DISEASE BY "SHUTTLE KISSING."

Notwithstanding the awakening conscience of the nation at large in matters of elementary hygiene, there are too many disgusting and dangerous habits and practices still tolerated which have been bequeathed from less fastidious and less sanitary generations of the past. One of these is known as "shuttle kissing," a process of threading in the weaving sheds by placing the shuttle to the lips. In this abomination the shuttle may be "kissed" by two or even more persons, not once or twice but repeatedly. The Local Government Board instituted an inquiry into the possibilities of transmission of disease in this way, and have now published the report of the committee appointed for the purpose. It is concurred in by all the members that the practice is objectionable; but the recommendations betray a certain lack of backbone which is unsatisfactory. Thus they do not advise regulations to prevent the "kissing"; but express a pious hope that newer forms of shuttle in which it is unnecessary may be adopted. At the same time they deprecate the practice "on the ground simply of decency and cleanliness" apart from the medical or public health aspect. How these two aspects can possibly be separated, and why cleanliness should thus be divorced from public health is not vouchsafed to us. Probably the medical member of the committee had to water down his own views in order to secure the concurrence of his lay co-investigators.

### AN EXPERIMENT IN INSTITUTIONAL LAUNDRIES

The experimental management of Park Hospital Laundry for one year on what are described as business methods has been advocated at a meeting of the Managers of the Metropolitan Asylums Board in a report of the Laundry Sub-Committee. Mr. H. Spender referred to the proposal to save £8 a week by lowering wages, but took exception to it on the ground that it would casualise women's labour, with the inevitable result of degrading it. Briefly, it is hoped to induce those who have passed through the schools to accept positions in the hospitals and other institutions by the training which will be carried on, and that in this way skilled labour will replace the unskilled labour which is now employed at considerable expense. After some discussion the committee's recommendations were carried. It should be added that Professor Smith said that the committee would not impose on children certain disgusting forms of labour which critics of the scheme had alleged to be in view. Coming as the Professor's first public utterance after his election as vice-chairman of the Board, we must urge him in the light of this promise to make it absolutely clear what are the precise forms of labour which he seeks to have taught to the children, as want of definition in this regard might well make the best intentions, especially if economy in wages be aimed at, mistaken for impositions of an undesirable kind. Professor Smith's chief, by the way, for the ensuing year is Mr. Walter Dennis, who was re-elected chairman at the same meeting. From every point of view we urge Mr. Dennis and Professor Smith to allow hospitals and institutional managers to share their experience by publishing a report on the outcome of the scheme.

### THE ALLEGED SCANDAL AT WOOLWICH.

Four weeks ago we had to draw attention to a specially called meeting, over which the Mayor of Woolwich presided, at which grave charges were preferred against Mr. Woodford, the secretary of the Woolwich and Plumstead Cottage Hospital. A resolution was proposed calling on the committee and the secretary to resign in May, in order that a special meeting of subscribers might elect their successors. The resolution was carried with one dissentient. Since then considerable excitement has prevailed, and statements of a lively character have been made, as, for instance, at the recent meeting at St. John's Schools, when the motion of the rector, the Rev. A. M. Pickering, that the committee should resign at the annual meeting, was lost. Mr. Mortimer was then, however, appointed temporary secretary; a visiting committee has been appointed, and a requisition book established. In addition, a subcommittee of five to consider the general management and expenditure has been appointed, consisting of the Rev. C. A. Berry, Mr. Nicholls, Mr. Mitchell, Mr. Kimber, and Mr. Pitt. But what has become of the Charity Commissioners, who were alleged by Mr. Kimber to have had the matter before them a month ago?

### A SOUTH AFRICAN MILLIONAIRE'S DEATH.

The death of Sir Julius Wernher, head of the South African house of Wernher, Beit and Co., which took place last Tuesday, comes only a fortnight after The Hospital had been able to announce the acceptance by the Institute of Bankers of the fine sanatorium, near Wokingham, from the Wernher-Beit firm. Sir Julius was born at Darmstadt in 1850, and the story of his great wealth is part and parcel of the race for the mines at Kimberley, which was the chief financial landmark of the seventies and eighties.

### A SURPRISE TUBE.

Some two hundred subscribers and friends of the General Hospital at Tunbridge Wells assembled on Wednesday last week to honour a very generous supporter of the institution. Since the discovery of the uses of $x$-rays and other advances in electrical science to aid the diagnosis and treatment of disease, Sir David L. Salomons, Bart., has taken a deep interest in the electrical department at the Tunbridge Wells Hospital, and from time to time, as improvements were made in the apparatus, has kept the department well up to date by his gifts. For instance, the room in which the electrical plant was installed has proved too small for the increase of work, and on learning this he generously offered to defray the cost of an extension. This he came on Wednesday last week formally to open. Lady Salomons and Miss Salomons accompanied Sir David, and were received by the chairman of the hospital, Mr. F. Wadham Elers, and many members of the committee. The honorary radiographer, Mr. G. W. Howard, was also present and demonstrated the apparatus, which consists of two $x$-ray plants, one in each room, the smaller set being chiefly for treatment and screening. There is also a Finsen light, apparatus for cautery, galvanism, faradisation, ionisation, and so on, the current for which comes from the main supply of the town. After a brief opening ceremony Sir David read a paper upon "Radium," tracing its discovery and subsequent uses in a lucid manner, which was followed with great attention as the properties of radium were gradually explained. To the vote of thanks, proposed by the treasurer and seconded by the senior physician of the hospital, Sir David replied, and to the surprise of those present, handed a tube to the treasurer containing .01 gramme of pure radium bromide. It is intended for the use of patients of the General Hospital, and, upon certain conditions, for the use of patients of medical men outside the hospital. As may be imagined, this further presentation of radium was very gratefully accepted.

### LORD WANDSWORTH'S BEQUEST ALLOCATED.

Sir William H. Bennett has decided to allot the whole of the £10,000, entrusted to him by the late Lord Wandsworth for the promotion of medical research, to the London School of Tropical Medicine, under conditions which include the establishment of a Research Scholarship tenable for two or three years, to be given by preference to a British subject. The Committee of Management of the Seamen's Hospital have been appointed by Sir William to be the trustees of the fund; and the scholar will be appointed by that body on the recommendation of the Committee of the London School of Tropical Medicine. The fact that Sir W. H. Bennett is senior surgeon to the Seamen's Hospital has no doubt predisposed him to this application of Lord Wandsworth's bequest; and the object is certainly a most worthy one. The first Wandsworth scholar will make human blood parasites the prime objects of his study, and will proceed to the West Coast of Africa for the purpose. If the whole of the bequest is applied to this single scholarship, nearly £400 a year would attach to it. In any case it may be well to point out that the London School of Tropical Medicine will still need much more financial assistance than this to carry out its work to the greatest advantage of the Empire, and with similar advantages to that which the open-handedness of Liverpool merchants has secured to the sister school in that city.

### X-RAY WORK IN A POOR-LAW INFIRMARY.

When presenting his annual report to the Wandsworth Board of Guardians at the last Board meeting, the Medical Superintendent, Dr. Miller, stated that in October last there had been established an *x*-ray department, and that upwards of fifty cases had been treated. They are also equipping a room at their institution, the St. James' Infirmary, with some of the latest apparatus for electrical therapeutics. He made especial reference to the assistance which he had received from Mr. W. E. Kinsman, M.P.S., Pharmacist to the Infirmary, who, after devoting much time and study to the science, Dr. Miller stated to be fast becoming an expert radiographer.

### "PEACEFUL PICKETING" IN THE PROFESSION.

Under this heading four weeks ago we referred to an incident at Bristol which led to the non-appointment of a lady medical inspector, owing to the applicants having received a warning notice from the British Medical Association not to accept the post, on the ground that the salary offered was inadequate to the work. Whether this were the case or not we did not then inquire, our point merely being to remind a member of the Bristol Health Committee, Mr. H. Anstey, who objected to the "warning" of the applicants by the British Medical Association in the office, that "it is not easy for any professional organisation to find out who are candidates for any post except by attending on the day of application." To this point of view we adhere. But in justice to the Bristol Health Committee it should perhaps be added that the whole matter is a storm in a teacup, one of those irritating mischances that are almost impossible to avoid. The matter really turns upon a badly worded advertisement. Without the actual words before us, we think it is a fair summary to say that candidates were asked not merely to attend at fixed hours on certain days a week, but to keep in touch with the office. This latter request is understood to have caused all the trouble. It was argued that the phrase implied a working week of six days: on which interpretation "a protest" against the salary offered may have been not unreasonable. For the sake of the comity between the profession and the Bristol Health Committee we trust that if this badly worded advertisement be admitted by one side, the warning notice will be withdrawn by the other.

### PHARMACISTS' OBJECTION TO SERVE ON JURIES.

The National Union of Assistant Pharmacists holds its fourth annual meeting on Whit-Monday in Birmingham. Mr. J. Wilson, M.P.S., Pharmacist to the Birmingham Dispensary, is President. Amongst the resolutions to be discussed in a lengthy agenda we observe this: "That wherever medicines are compounded or dispensed, such place shall be subject to the inspection under the Acts of Parliament which govern the sale of drugs. The distribution of medicines to be deemed a sale for the purposes of these Acts." We are not sure of the true importance of this association passing this resolution, but, were the principle adopted, there would be many a radical change necessary in institutions and in doctors' surgeries. The Union is also seeking that exemption from jury service be extended to include all practising pharmacists, and has petitioned the Select Committee, which is considering the entire question of jury service, for this extension.

### POOR-LAW DISPENSERS AND THEIR SALARIES.

Quite recently the dispensers in his Majesty's Prison Service received an improvement in their conditions of service. Their hours were reduced, and they were raised to the status of second-class clerks, which gives an improved allowance when travelling on duty. They were also accorded the title of "pharmacist" in place of "compounder," the former being significant of their qualification. Now we learn that the Poor-Law dispensers are memorialising the Local Government Board for an increase in their rate of pay. The present rate is from £120 to £180 per annum, rising by increments of £10 every two years. This applies only in London; in the provinces the pay is often higher, some receiving salaries on a scale rising to £250. As the cost of living is higher in London than in most places, it seems that the Metropolitan dispensers have a grievance. We have often given examples of the ability of the Poor-Law dispenser, and appreciate the necessity of making the salary sufficiently attractive to retain the best men. The signatures to the memorial are being collected by the Public Pharmacists' Association, a body which is showing much activity.

### "FOR THE PROMOTION OF CRUELTY TO ANIMALS."

That the words in the heading of this paragraph were called out by some of those who attended the eighty-eighth annual meeting of the Society for Prevention of Cruelty to Animals, as a substitute, last Tuesday, gives an indication not of the levity of those present but of the inflamed feelings that were aroused. The vivisection controversy apparently loses nothing in animosity for all the findings and recent publication of the Royal Commission's Report. Suffice it to say that the excitement arose out of the Hon. Stephen Coleridge's criticism of a resolution of the Council which had been embodied in the report, to the effect "That they claim and allow perfect freedom of opinion to all the members of the society on the question of vivisection." The report was adopted on a recount, following on a show of hands, by 91 votes to 54. The anti-vivisectionists lost 12 votes on the final voting, and those in favour 4. An amendment not to re-elect Lord Cheylesmore and Mr. Stockman to the Council was defeated. On the whole, perhaps, the best cause never loses by a strong opposition: a hard saying, but one which the lives of far-seeing statesmen have approved.

### OLD RESIDENTS' CLUBS IN THE PROVINCES.

Mr. James Phillips, F.R.C.S., hon. surgeon to the Bradford Royal Infirmary, as President of the Leicester Infirmary Old Residents' Club for the year, took the chair at the second anniversary dinner last week. The good muster of old residents was increased by the attendance of the honorary medical and surgical staff of the infirmary. Interesting speeches enlivened or pointed by reminiscences were made by Dr. Pratt, the senior physician, Mr. Bond, the senior surgeon, Dr. Astley Clarke, Mr. H. J. Blakesley, F.R.C.S., Dr. Frank Hinds, of Worthing, Mr. Garnett Wright, F.R.C.S., of Manchester, Mr. M. H. Phillips, F.R.C.S., of Sheffield, and others. Dr. C. A. Moore was then elected president for the ensuing year, and it was decided to arrange for the annual dinner to take place at Leicester. This is an important decision, for though annual dinners at hospitals with training schools are the custom, it is probably unique for a provincial general hospital to have its Old Residents' Club. At any rate, suggestions were made for the compilation of a complete list of former residents with a view to their closer association with the infirmary. Mr. Garnett Wright is the hon. secretary.

### HEALING THE SICK BY ACT OF PARLIAMENT.

Dr. Owen Coleman, who only eight weeks ago resigned the post of senior medical officer to Surbiton Cottage Hospital after having held it for about forty years, has given some curious figures to the world on the death rate and birth rate of Surbiton. As medical officer of health, Dr. Coleman remarks the lowest birth rate (18.5 per thousand) which he has ever had to record. Compared with Surbiton, London can show a birth rate of 25, and England and Wales one of 24.4, so that when noting the death rate of 8.9 (which rises two points on correction as the net rate), Dr. Coleman has to conclude that though Surbiton's death rate be low, its birth rate is almost phenomenally low also. It is interesting once more to record the professional opinion that, as far as legislation which concerns preventive and health measures at any rate, the old political cliché "You cannot make a man sober by Act of Parliament" is utterly discountenanced and discredited. In acknowledging a vote of thanks, Dr. Coleman pointed out that "the increase in legislation for the purpose of enforcing sanitary measures had resulted in a great improvement in health." We have always maintained that a large share of future triumphs in medicine will belong to an extended public health service for the enforcement of preventive measures.

### HOSPITAL APPOINTMENTS IN MELBOURNE.

The appointments to the honorary medical and surgical staffs of the metropolitan hospitals in Melbourne are now nearly all made on the recommendation of an Advisory Board, which includes members of the committee of the institution concerned and an equal or smaller number of representatives of the University, the latter including members of the Faculty of Medicine. This new system has, on the whole, worked satisfactorily, and is far and away preferable to the election of honorary staffs by the votes of the contributors to the hospitals, a system in vogue till comparatively recently at the Melbourne and Women's Hospitals. There seems a fear, however, that the University influence is becoming too strong. Two members of the Advisory Board, Professor R. J. A. Berry and Dr. James W. Barrett, have advised that, in view of the development of the Alfred Hospital, candidates for surgical appointments should hold the F.R.C.S. Eng. or the Mastership of Surgery of the University of Melbourne, and that the surgeons and assistant surgeons and physicians and assistant physicians should devote themselves entirely to the practice of surgery or medicine respectively. We understand that members of the Victorian Branch of the British Medical Association are up in arms against the action of these two gentlemen, and have passed a resolution requesting the Alfred Hospital Committee to hear the views of their Council before taking any action. We may remind English hospitals that there are comparatively few practitioners in Melbourne who adhere strictly to surgery, for instance, and would refuse to treat a medical case that came in their way.

### A LIVERPOOL CHAIRMAN AND THE INSURANCE COMMISSION.

Mr. H. Wade Deacon, chairman of the Liverpool Royal Infirmary and of the Liverpool Voluntary Aid Council, has been invited to become one of the representatives of hospitals on the National Health Insurance Commission (England).

### THIS WEEK'S DRUG MARKET.

The most important event of the week in the Drug market is a substantial advance in the price of potassium, sodium, and ammonium bromides. The advance had been expected so long that many purchasers had grown tired of waiting for it, and had ceased to hold large stocks; now that the rise in price has at last been announced they find themselves unprepared for it. Opium continues its downward tendency, although the actual reduction in price is at present small; morphine also continues to tend downwards in price, and apomorphine is materially cheaper. The demand for quinine, which had quieted down considerably at the end of last week, has revived again, and a fair business has been done at the lately advanced price. The demand for cod-liver oil is extremely quiet, and prices tend lower if anything, although there is not much room for any noteworthy reduction. Seidlitz powder is slightly lower in value, as also is tartarated soda. Buchu leaves are rather dearer. Sugar of milk is again lower in price.

### TO OUR READERS.

Contributions are specially invited from any of our readers to these columns. They should deal with topical subjects and news. They must be authenticated for the information of the Editor only. The minimum payment if published is 5s. There is no hard-and-fast rule as to space, but notes of about twenty lines in length are preferred.

